# pH-Responsive Properties of Asymmetric Nanopapers of Nanofibrillated Cellulose

**DOI:** 10.3390/nano10071380

**Published:** 2020-07-15

**Authors:** Maud Chemin, Baptiste Beaumal, Bernard Cathala, Ana Villares

**Affiliations:** French National Research Institute for Agriculture, Food and Environment (INRAE), UR Biopolymer, Interactions, Assemblies (BIA), F-44316 Nantes, France; maud.chemin@inrae.fr (M.C.); baptiste.beaumal@gmail.com (B.B.); bernard.cathala@inrae.fr (B.C.)

**Keywords:** nanocellulose, cellulose nanofiber, film, actuator, bending

## Abstract

Inspired by plant movements driven by the arrangement of cellulose, we have fabricated nanopapers of nanofibrillated cellulose (NFC) showing actuation under pH changes. Bending was achieved by a concentration gradient of charged groups along the film thickness. Hence, the resulting nanopapers contained higher concentration of charged groups on one side of the film than on the opposite side, so that pH changes resulted in charge-dependent asymmetric deprotonation of the two layers. Electrostatic repulsions separate the nanofibers in the nanopaper, thus facilitating an asymmetric swelling and the subsequent expanding that results in bending. Nanofibrillated cellulose was modified by 2,2,6,6-tetramethylpiperidin-1-yloxyl radical (TEMPO) oxidation at two reaction times to get different surface concentrations of carboxylic acid groups. TEMPO-oxidized NFC was further chemically transformed into amine-modified NFC by amidation. The formation of graded nanopapers was accomplished by successive filtration of NFC dispersions with varying charge nature and/or concentration. The extent of bending was controlled by the charge concentration and the nanopaper thickness. The direction of bending was tuned by the layer composition (carboxylic acid or amine groups). In all cases, a steady-state was achieved within less than 25 s. This work opens new routes for the use of cellulosic materials as actuators.

## 1. Introduction

In plant cell walls, cellulose fibrils are embedded in a highly swellable matrix consisting of hemicelluloses, pectins, structural proteins and/or lignins. This composite structure confers each cell a stable shape, the ability to develop high turgor pressures, and remarkable mechanical properties. The structure of cellulose fibers gives mechanical properties to the plant, and their organization and alignment play an important role in complex plant functions [1,2,3,4,5]. For example, in wild wheat, the arrangement of cellulose fibrils drives seed dispersal. The cellulose microfibrils are well aligned along the awn axis in the cap while they are randomly oriented at the ridge. These two cellulose arrangements (aligned and random) expand differently depending on ambient humidity conditions. Therefore, changes in humidity cause asymmetric swelling and bending of the awns, thus dispersing the seeds [2]. Similarly, in the case of pines, the scales of seed-bearing pine cones also move in response to changes in relative humidity. When the air is dry the scales gape open, releasing the cone’s seeds, and they close up as the air is damp [3].

In cellulose, water uptake is driven by the high number of accessible hydroxyl groups and the presence of surface charge [6]. In previous works, Falt et al. [7] demonstrated that swelling was directly related to the fibers surface charge. Thus, carboxymethylated cellulose, which contains negatively charged carboxymethyl groups on the surface (pKa = 4.2), swelled in higher extent in water than unmodified cellulose. Indeed, when increasing pH, carboxymethyl groups deprotonate and swelling is boosted by the repulsion between charged groups. Similarly, Olszewska et al. [6] observed that cationic cellulose nanofibrils swelled at low pH due to the protonation of the amine groups, and they concluded that the higher the charge of nanofibrillated cellulose (NFC), the more water uptake. 

Inspired by plant movements driven by cellulose swelling and deswelling, Wang et al. [8] fabricated nanofibrillated cellulose (NFC) films showing humidity-controlled reversible actuation. Thin NFC films bent upon exposure to relative humidity from 10 to 40%. The authors ascribed the actuation behavior to the formation of bilayer-like structure consisting of asymmetric humidity-swollen and non-swollen layers. Kuang et al. [9] took advantage of the dehydration in cellulose for fabricating soft actuators consisting of aligned NFC onto a passive layer. In this case, bending was driven by asymmetric water evaporation, and actuation was tuned by temperature and nanofiber alignment. The nanometer lateral diameter of NFC favored bending compared to paper fibers [10], which revealed the important role of nanometric sized materials. NFC has a high aspect ratio (3–5 nm in width and upwards to several micrometers in length), and exhibits high stiffness (138 GPa) and low density (1.6 g cm^−3^) [11,12,13]. These features explain the usefulness of NFC materials as reinforcement components [14,15]. Gradient composite materials containing nanocelluloses enriched layers at different ratios show enhanced mechanical properties [16,17]. Hence, elastic properties and ductility of nanopapers composed of NFC and tailor-made synthetic copolymers can be modulated by the ratio of both components [18].

In this work, we take advantage of pH-responsive properties of modified NFC to fabricate actuators. We investigated the induced actuation behavior by the fabrication of asymmetric NFC nanopapers containing different charge concentrations along the film thickness. Asymmetry was therefore achieved by the successive deposition of NFC layers containing different degrees of functionality (graded films) or different chemical groups. We focused on pH-responsive properties provided by carboxylic acid groups or amine groups introduced at the surface of NFC.

## 2. Materials and Methods

### 2.1. Materials and Reagents

(2,2,6,6-tetramethylpiperidin-1-yl)oxyl radical (TEMPO), sodium chlorite (NaClO_2_), sodium hypochlorite (NaClO), hexamethylenediamine, *N*-(3-dimethylaminopropyl)-*N*’-ethylcarbodiimide hydrochloride (EDC), *N*-hydroxysuccinimide (NHS), potassium hydroxide (KOH), sodium acetate, sodium hydroxide (NaOH) and hydrochloric acid (HCl) were purchased from Sigma-Aldrich (France) and were used without further purification. A Spectra/Por dialysis membrane, MWCO 12–14,000 Da, was purchased from Spectrum Laboratories Inc. (Piscataway, NJ, USA). Water was purified by the Millipore Milli-Q purification system (18.2 MΩ).

Nanofibrillated cellulose (NFC) was provided by Centre Technique du Papier (CTP), Grenoble (France). NFC was prepared from bleached pulp paper submitted to endoglucanase treatment, and homogenized at an operating pressure of 1500 bar (4 passes). NFC was purified by filtration (1–1.6 µm).

### 2.2. TEMPO Oxidation

NFC (6 g) was suspended in sodium acetate (0.1 M) at 40 °C. The TEMPO radical (0.7686 g) and NaClO_2_ (8.174 g) solubilized into sodium acetate were added to the NFC dispersion. The TEMPO-mediated oxidation started by adding NaClO (0.538 g) under stirring at 40 °C [19]. Two different reaction times were used, 24 and 48 h, with the aim of having different oxidation degrees of TEMPO-oxidized cellulose nanofibrils (TOCN) (TOCN5 and TOCN9, respectively). TEMPO-oxidized NFC was purified by filtration (1–1.6 µm) and then dialyzed (molar mass cut off 12–14,000 Da) against Milli-Q water for 15 days.

### 2.3. Amidation

The carboxylic acid groups from the TEMPO-oxidized NFC were transformed into amine groups as previously described [20,21]. An 8 g L^−1^ suspension of TEMPO-modified NFC (150 mL, pH 7) was degassed by bubbling nitrogen for 20 min, and 0.6 mmol of NHS were added, followed by 0.6 mmol of EDC, and the pH was adjusted to 6.5 by the addition of several droplets of HCl 0.1 M. After stirring for 30 min, 2.4 mmol of hexamethylenediamine were added and the pH of the reaction mixture was adjusted to 9.2 with KOH 0.1 M. The reaction was sonicated (7 W at 20 kHz for 60 s, with probe diameter 3.2 mm, QSonica Sonicator) and incubated under stirring at room temperature overnight. Amine-functionalized NFC (AMCN) was purified by centrifugation (9500 g, 30 min, 20 °C) and then dialyzed (molar mass cut off 12–14,000 Da) against Milli-Q water for 15 days.

### 2.4. Characterization 

#### 2.4.1. FT-IR

Infrared spectra were obtained from KBr pellets containing freeze-dried NFC samples placed directly in a Nicolet iS50 FT-IR spectrometer (Thermo Scientific, France) in absorbance mode. All spectra were collected with a 4 cm^−1^ resolution after 200 continuous scans from 400 to 4000 cm^−1^. TEMPO-oxidized NFC was acidified to pH 2 by the addition of HCl in order to protonate carboxylic acid groups.

#### 2.4.2. Conductometry

The quantity of charges on the NFC surface was measured by conductometric titration with 0.01 M NaOH or HCl solutions for TEMPO-oxidized or amine-modified NFC respectively, by a TIM900 titration manager and a CDM230 conductimeter equipped with a CDC749 conductivity cell (Metrohm, France). The degree of oxidation (*DO*) or substitution (*DS*) was calculated from the following equation [22]:

(1) $$DO/DS=\frac{162\left(V_{eq2}-V_{eq1}\right)C}{m-M\left(V_{eq2}-V_{eq1}\right)C}$$

where *V*_*eq*1_ and *V*_*eq*2_ are the equivalence volumes of NaOH or HCl (L), *C* is the NaOH or HCl concentration (mol L^−1^), *m* is the dried weight of NFC (g), *M* is the molecular weight of the carboxylic acid group or the amine functionality, and 162 is the molecular weight of anhydroglucose (g mol^−1^).

### 2.5. Nanopaper Preparation

TOCN dispersions were set at pH 2 and AMCN at pH 12, in order to protonate the COOH and deprotonate the NH_2_ groups, respectively. Nanopapers were prepared by filtration of NFC dispersions at 10 g L^−1^ through polyvinylidene fluoride (PVDF) membranes (pore size 0.22 µm). Filtration was performed at 50 mbar during 30 min for the first layer, and at 300 mbar during 60 min followed by 30 mbar during 90 min for the second layer. Then, nanopapers were dried at ambient temperature in a desiccator under a weight of 300 g. Nanopaper thickness was measured by a micrometer, and density was calculated by weighing the films. The nanopaper charge was calculated taking into account the amounts of TOCN or AMCN used for the film fabrication and the dimensions of the nanopaper.

### 2.6. Bending Curvature Analysis

Nanopapers were first immersed in water in order to discard any effect from water uptake. Then, they were immersed in NaOH or HCl 0.1 M, and the bending curvature was determined by recording movies during the pH variation, and periodically extracting pictures. Each nanopaper was fixed with a binder clip leaving a free length of approximately 20 mm. Image analysis was performed using ImageJ^®^ software. Curvature was evaluated from the measurement of the X-Y coordinates. Film bending was assumed to be an arc of a circle, and the curvature was measured by fitting the time evolution to a circumference function, as previously described [23]. The bending curvature (*κ*) was calculated from the radius (*r*) of the fitted circle according to the following expression:

(2) $$\kappa =\frac{1}{r}$$

All data expressed are the average of at least 3 measurements realized on two different films.

## 3. Results and Discussion

### 3.1. Preparation of Cellulose Nanofibers at Different Degrees of Substitution

**Figure 1:**
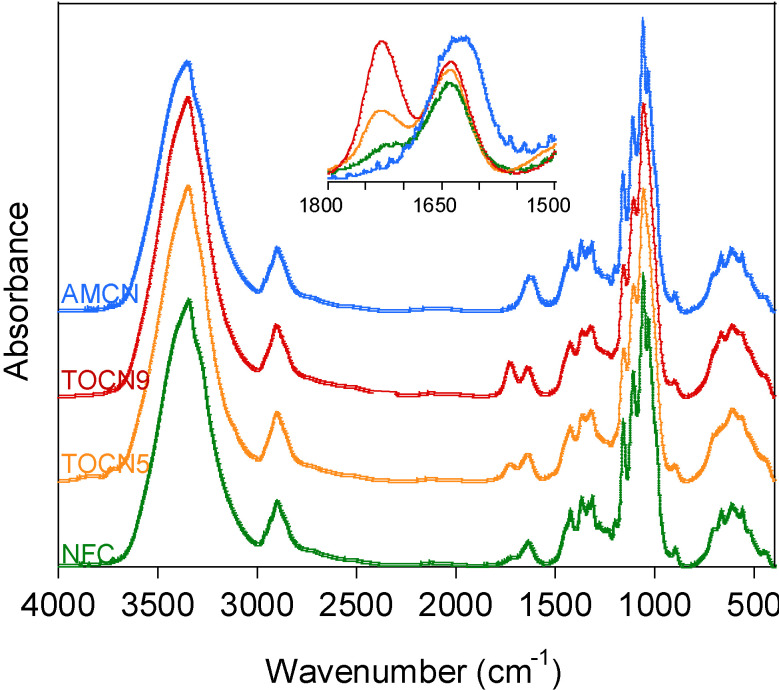
FT-IR spectra in absorbance of the unmodified nanofibrillated cellulose (NFC), TEMPO-oxidized NFC (TOCN5 and TOCN9) and amine-modified NFC (AMCN). The inset shows the 1800–1500 cm^−1^ region.

Cellulose nanofibers were modified by the selective oxidation of the primary hydroxyl groups to carboxyl groups by the (2,2,6,6-tetramethylpiperidin-1-yl)oxyl radical (TEMPO) [24]. TEMPO oxidation has been demonstrated to be time-dependent [19]; therefore, two oxidation times (24 h and 48 h) were chosen for obtaining two different degrees of oxidation (*DO*). Then, the carboxyl groups of TOCN5 were further modified by amidation with hexamethylenediamine catalyzed by the NHS-EDC system [20] to obtain amine-modified cellulose nanofibrils (AMCN). Chemical modifications were confirmed by FT-IR (Figure 1). TEMPO-modified NFC showed a strong band at 1730 cm^−1^ corresponding to protonated carboxylic acid groups (C=O stretching) [22]. As expected, the intensity of the band increased with the *DO*. Then, when TOCN5 was transformed into AMCN, the carboxylic acid band at 1730 cm^−1^ disappeared, indicating the complete conversion of COOH groups, and the typical amide I band (C=O stretching) was detected at 1645 cm^−1^ [25]. These bands did not appear for unmodified NFC, thereby indicating the chemical modifications in TOCN and AMCN.

Conductometric titration was used to quantify the negative surface charge due to the carboxyl groups of the two TEMPO-oxidized cellulose nanofibrils (TOCN). The TEMPO oxidation performed during 24 h resulted in 0.301 mmol g^−1^ (TOCN5) whereas longer oxidation times (48 h) gave 0.531 mmol g^−1^ (TOCN9), which corresponded to *DO* 0.05 and 0.09, respectively (Table 1). Conductometric titration curves are shown in Figure S1. The positive charge was titrated with HCl and was slightly lower than TOCN5 (0.227 mmol g^−1^).

### 3.2. Fabrication of pH-Responsive Nanopapers

Nanopapers were prepared from aqueous dispersions of NFC by vacuum filtration at controlled pressure, followed by drying in a desiccator at room temperature. Disc-shaped films (diameter of 36 mm) were cut into two rectangles of 7 × 24 mm. The nanopaper thickness was rather homogeneous for all the samples and equal to 50 ± 6 µm. We designed bilayer films charged differently in each layer in order to prepare graded nanopapers by the successive filtration of various combinations of NFC, TOCN5, TOCN9 and AMCN.

**Figure 2:**
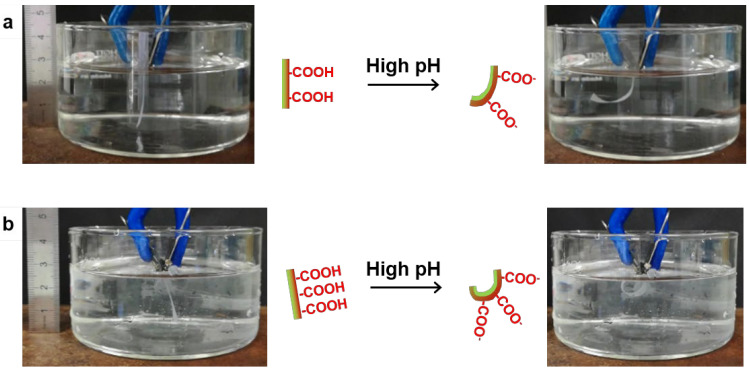
Photographs showing the bending of (**a**) NFC/TOCN5 and (**b**) NFC/TOCN9 nanopapers when they were immersed in high pH aqueous solutions (pH 13).

Firstly, we varied the amount of negative charges, i.e., the resulting nanopapers contained a higher concentration of charged groups on one side than on the opposite side, so that pH changes resulted in charge-dependent asymmetric deprotonation of the two layers. Electrostatic repulsion separated the nanofibers in the charged layer, thus facilitating an asymmetric swelling of the nanopaper that resulted in bending. This effect was clearly demonstrated by comparing the response to pH of NFC/TOCN5 and NFC/TOCN9 nanopapers. When nanopapers were first immersed in water, generally, they were straight and water uptake did not modify their bending. In some cases, nanopapers bent initially in water; however, after several seconds, they returned to the straight position. Contrarily, when nanopapers were immersed in NaOH 0.1 M, they changed their shape from straight (time 0) to bent, reaching a maximum curvature over 30 s (movies S1 and S2 provide supporting information for NFC/TOCN5 and NFC/TOCN9 nanopapers, respectively). Figure 2 shows the photographs of the nanopapers when they were immersed in NaOH 0.1 M (pH 13) at time 0 and after 60 s. Alkaline pH deprotonated the COOH groups from both TOCN5 and TOCN9 layers, creating negative charges, whereas the NFC layer remained neutral, which caused the asymmetric swelling and bending. As shown in the photographs, the extent of bending was then clearly influenced by the charge concentration.

**Figure 3:**
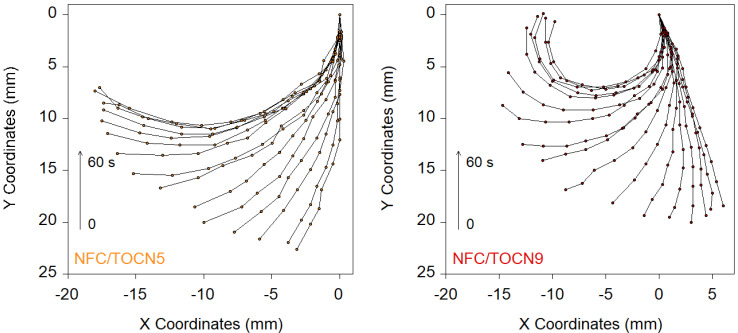
X-Y coordinate plots showing the evolution of the bending of NFC/TOCN nanopapers when they were immersed in high pH aqueous solutions (pH 13) for times from 0 to 60 s.

The curvature of the bilayer nanopapers was evaluated from the measurement of the X-Y coordinates of the samples during the bending process. Figure 3 shows the X-Y coordinates of the films at different times during bending for the NFC/TOCN5 and NFC/TOCN9 nanopapers.

**Figure 4:**
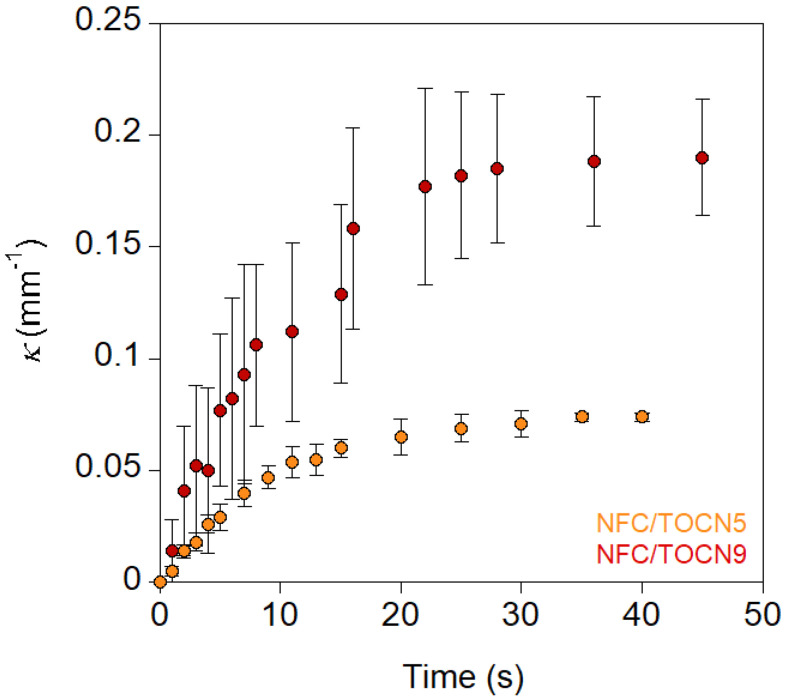
Curvature of the NFC/TOCN nanopapers as a function of time when they were immersed in high pH aqueous solutions (pH 13).

Bending was assumed to be an arc of a circle, and the curvature was therefore measured by fitting to a circumference, whose radius can be calculated (see Experimental section and Figure S2) [23]. Figure 4 shows the curvature values (*κ*) of NFC/TOCN5 and NFC/TOCN9 nanopapers as a function of time. In both cases, curvature underwent a rapid increase followed by a plateau in which maximal curvature was reached in less than 20 s. Increasing the *DO* from 0.05 to 0.09 significantly modified the actuation behavior, resulting in higher maximal curvatures.

Similarly to the water adsorption and desorption kinetics [9], the changes in nanopaper curvature as a function of time can be described by a pseudo-first order kinetic model [26], expressed as:

(3) $$\frac{d\kappa }{dt}=k_{1}\left(\kappa_{max}-\kappa \right)$$

The integration of the above equation results in the following expression:

(4) $$\kappa =k_{1}-\kappa_{max}e^{-k_{1}t}$$

where *κ* is the nanopaper curvature as a function of time (*t*), *κ_max_* is the maximum curvature, and *k*_1_ is the actuation rate constant.

Maximum curvature and the actuation rate constant were calculated by fitting to the pseudo-first order kinetic equation. The actuation rate was determined from the initial slope of the curvature as a function of time, as shown in Figure S2. Table 2 reviews the nanopaper thickness (*d*), charge, pH stimulus, actuation time (*t_a_*), maximum curvature (*κ_max_*), actuation rate constant (*k*_1_), and actuation rate (*dκ/dt*) for the NFC/TOCN nanopapers studied.

When the charge concentration increased, both the maximum curvature and the actuation rate increased; however, this impact of charge concentration on *κ_max_* and *dκ/dt* was not proportional. Thus, an increase of 1.5 in charge resulted in a three-fold increase in *κ_max_* and a two-fold increase in *dκ/dt.* Actuation rate constants were similar for both NFC/TOCN5 and NFC/TOCN9 nanopapers. Actuation was very fast, hence, the maximum curvature was reached in only 12 and 15 s for NFC/TOCN5 and NFC/TOCN9 nanopapers, respectively. These results were in agreement with humidity responsive NFC actuators, which achieved maximal curvatures in 11 s [8]. When more complex actuators are investigated, such as hygromorph composites of flax/maleic anhydride grafted polypropylene, the time for reaching maximal curvature increases from minutes up to several hours, depending on the actuation conditions (relative humidity) [23].

**Figure 5:**
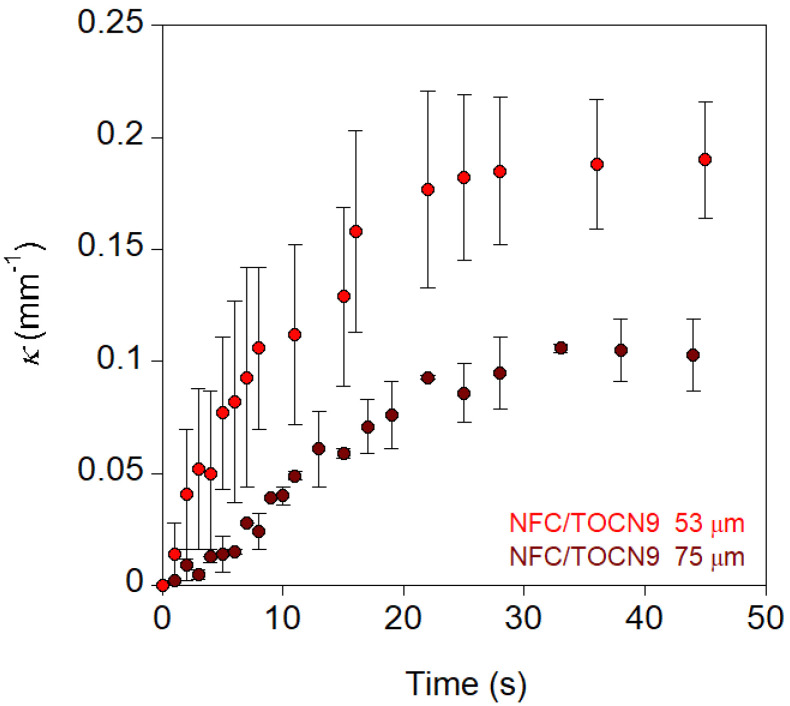
Curvature as a function of time of the NFC/TOCN9 nanopapers with thicknesses of 53 and 75 µm when they were immersed in high pH aqueous solutions (pH 13).

Wang et al. demonstrated that bending curvature became reduced when the film thickness increased [8]. To quantitatively verify the impact of thickness (*d*), NFC/TOCN9 nanopapers were prepared by adjusting the amounts of NFC and TOCN9 to obtain a thicker film containing a dried mass of 85.2 ± 1.6 mg and displaying a thickness of 75 ± 1 µm. In comparison, the previously described nanopapers had a dried mass of 59.6 ± 4.3 mg and a thickness of 53 ± 2 µm. The bending of thick nanopapers is shown in Figure S3. Figure 5 shows the evolution of curvature as a function of time for the NFC/TOCN9 nanopapers, with different thicknesses immersed in NaOH 0.1 M (pH 13).

The thinner nanopaper (53 ± 2 µm) showed higher bending than the thicker (75 ± 1 µm) nanopaper, which confirmed the lower actuation response as the thickness increased. The maximum curvature and the actuation rate values both decreased when the film thickness increased even if the overall charge in the thicker nanopaper was higher (Table 2). Furthermore, the actuation rate constant was significantly lower for the thicker nanopapers, whereas the response time increased (23 s), which suggested a slower response compared to the thinner nanopapers. In their study, Wang et al. [8] prepared monocomponent NFC films and they found that only films with thickness less than 20 µm experienced high bending curvature when exposed to humidity. The increase in thickness to 38 µm exhibited only moderated curvature, and 48 µm resulted in a minimal curvature. Similar results were found by Zhang et al. for cellulose stearoyl esters films [27]. They observed an increase in the bending radius (and a decrease in curvature) when film thickness increased from 10.9 to 44.1 µm. The authors ascribed the lower curvature to higher stiffness of thicker films. In our study, the charge-driven asymmetric swelling allowed graded NFC/TOCN nanopapers to reach high curvature for thicknesses higher than 50 µm.

These results revealed that the key parameters for controlling actuation were the nanopaper thickness and the charge concentration. From the experimental data of NFC/TOCN nanopapers, a numerical model was developed to establish the relationship between the maximum curvature and the actuation rate as a function of the nanopaper thickness and charge:

(5) $$\kappa_{max}=0.352-6.18\times thickness\times density+0.074\times charge$$

(6) $$\frac{d\kappa }{dt}=0.028-0.420\times thickness\times density+0.003\times charge\;$$

**Figure 6:**
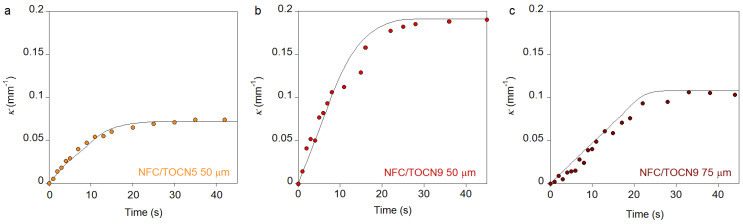
Curvature as a function of time of (**a**) NFC/TOCN5 (50 µm), (**b**) NFC/TOCN9 (50 µm), and (**c**) NFC/TOCN9 (75 µm) nanopapers when they were immersed in high pH aqueous solutions (pH 13). The circles correspond to experimental data and the solid lines represent the fit.

In these equations, thickness is expressed in mm, nanopaper density in g cm^−3^, and nanopaper charge in µmol. These two equations allowed modeling the curvature of NFC nanopapers in response to pH changes as a function of time. Figure 6 reviews the experimental data and the fit for the curvature plots.

In all cases, fitting results showed a good agreement with the experimental data, which validated the model used for these types of nanopapers in the studied conditions. The model considered that nanopaper thickness had a negative impact on both maximum curvature and actuation rate whereas charge clearly drove actuation.

**Figure 7:**
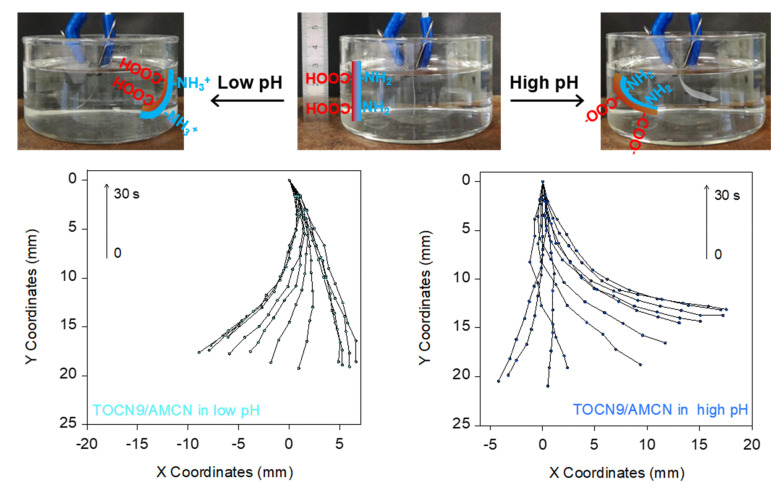
Photographs showing the change in bending of the TOCN9/AMCN films when they were immersed in low and high pH aqueous solutions (1 and 13, respectively); and X-Y plots showing the evolution of the curvature for times from 0 to 30 s.

Next, in order to increase the pH range of these NFC actuators, bilayer films containing TOCN9/AMCN were prepared. Depending on the pH, the resulting nanopapers contained negatively charged groups on one side (COO^−^), at high pH, and positively charged groups on the opposite side (NH_3_^+^), at low pH, so that the pH tuned the bending direction. Figure 7 shows the bending behavior when nanopapers were immersed in aqueous solutions either at pH 13 (NaOH 0.1 M) or at pH 1 (HCl 0.1 M). At high pH, carboxyl groups became deprotonated, which provided negative charge, whereas the amino groups were neutral. Therefore, while the TOCN9 layer was significantly swollen, the AMCN layer remained unchanged, causing asymmetric expansion and subsequent bending. In contrast, at low pH (pH 1), the carboxyl groups remained uncharged whereas the amino groups were positively charged thanks to their protonation. Therefore, the AMCN layer was swollen, and bending occurred in the opposite direction.

**Figure 8:**
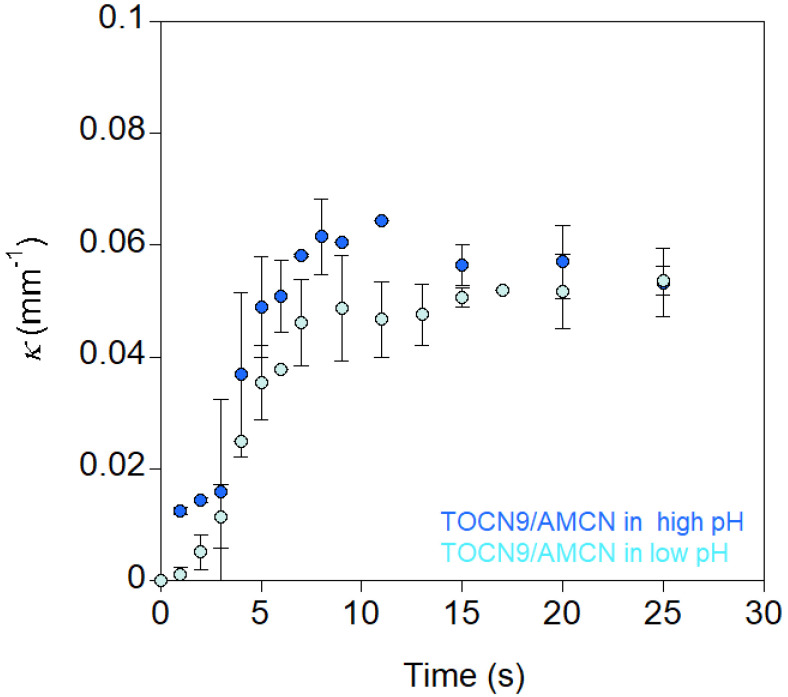
Absolute curvature as a function of time of the TOCN9/AMCN nanopapers immersed in low or in high pH aqueous solutions (pH 1 and 13, respectively).

When TOCN9/AMCN nanopapers were firstly immersed in water, some random films bent as if the TOCN9 layer became more swollen than the AMCN layer (some films bent and some films remained straight). Duan et al. [28] prepared bilayer hydrogel actuators containing chitosan (NH_2_) and cellulose/carboxymethylcellulose (COOH) layers cross-linked by epichlorohydrin. They observed that the films were in a straight state at pH 3.8. Above this pH, the cellulose/carboxymethylcellulose layer exhibited higher swelling because of the deprotonation of some carboxylic acid groups, resulting in expanding the cellulosic layer. Therefore, the bending observed after the immersion of TOCN9/AMCN nanopapers in water could be ascribed to the partial deprotonation of carboxylic acid groups from the TOCN9 layer in water. Thus, for monitoring the actuation of the TOCN9/AMCN nanopapers, they were directly immersed in acidic or alkaline aqueous solutions. The changes in curvature upon time are shown in Figure 8. The plots of curvature versus time show a sigmoidal behavior for the TOCN9/AMCN films, indicating a slow increase in curvature at the beginning of bending followed by a steep increase before reaching the maximal curvature. The low increase in *κ* observed between 0 and 3 s could be due to water uptake when nanopapers were immersed for the first time in the aqueous solutions at either high or low pH. Then, the curvature increased rapidly, corresponding to the formation of charged groups and the subsequent asymmetric swelling. Despite the different nature and concentration of charges, the absolute curvature reached similar values in both acidic and alkaline pHs (Figure 8), which indicated comparable bending deformation of both TOCN9 and AMCN layers.

Compared to NFC/TOCN nanopapers, TOCN9/AMCN nanopapers showed lower maximum curvature values in both acidic and alkaline pHs (Table 2 and Table 3 rows 1–2). Nevertheless, response was faster, which was reflected by the values of the actuation rate constants, which were significantly higher in the case of TOCN9/AMCN compared to NFC/TOCN nanopapers.

One of the hypotheses of the lower curvature values of TOCN9/AMCN nanopapers is the possibility of neutralization of the COOH and NH_2_ groups at the interface between the TOCN9 and AMCN layers, respectively. In order to prevent the interactions between COOH and NH_2_ groups, we prepared trilayer nanopapers composed of TOCN9, NFC in between and AMCN, and we determined the changes in curvature as a function of time (Figure S4). From the experimental values of curvature, the numerical model developed for the NFC/TOCN nanopapers was used for calculating the effective charge of TOCN9/AMCN and TOCN9/NFC/AMCN nanopapers (Table 4).

At low pH, maximum curvature and actuation rate were similar for TOCN9/AMCN and TOCN9/NFC/AMCN nanopapers despite their different theoretical charge concentrations (Table 4). Indeed, the effective positive charge for TOCN9/AMCN was lower compared to the theoretical value, confirming the neutralization of COOH and NH_2_ groups at the interface in the case of the bilayer nanopapers. Neutralization resulted in a loss of charge and therefore, the bilayer TOCN9/AMCN and the trilayer TOCN9/NFC/AMCN nanopapers presented similar effective positive charge concentration, which justified the similar *κ_max_* values. On the contrary, at high pH, actuation was significantly modified and the maximum curvature decreased from 0.062 mm^−1^ (bilayer) to 0.034 mm^−1^ (trilayer) which was partially explained by a decrease in theoretical charge values from 2.7 µmol (bilayer) to 1.7 µmol (trilayer). Furthermore, the high deviations of the effective negative charges from the theoretical values could not be entirely justified by neutralization at the interface, pointing at the deprotonation of a high number of COOH groups during the nanopaper preparation. Indeed, the TOCN9 layer was firstly deposited on the filter, then the NFC (in the case of the trilayer) and AMCN dispersions at pH 12 were added. Therefore, the alkaline pH from the AMCN dispersion might deprotonate some of the COOH groups, reducing the responsiveness of this layer. Consequently, the effective charge of the nanopapers would be lower than expected.

Despite the controllable responses obtained for all the nanopapers fabricated with NFC, TOCN and AMCN, no reversibility of the response was achieved. After the immersion in high or low pH solutions, nanopapers were again transferred to water; however, they remained curved. The advantage of using modified NFC was the intrinsic cohesion between layers that avoided using toxic cross-linkers such as epichlorohydrin. However, the absence of cross-linking between the nanofibers architectures may result in conformational changes during charge formation towards more stable arrangements. Then, when the stimulus was removed, and the charge disappeared, no rearrangement to the initial state occurred, and reversibility was therefore not achieved. Further studies are ongoing to attain reversibility for NFC nanopapers actuation.

## 4. Conclusions

This work demonstrates that graded NFC nanopapers are excellent candidates for the fabrication of actuators. Bilayer NFC nanopapers containing different amounts of surface charge have been prepared and the actuation behavior has been demonstrated to be influenced by the charge content. Thus, bending was more pronounced for films containing high charge TEMPO-modified NFC (0.53 mmol g^−1^) compared to low charge (0.30 mmol g^−1^). The reversal of charge from negative to positive by the introduction of amino groups resulted in the same bending but in the opposite direction. This work demonstrates the controlled bending of nanocellulose nanopapers and establishes the relationship between the nanopaper characteristics (charge and thickness) and the bending response to different pHs. The possibility of predicting the nanopaper response opens new routes for the fabrication of programmable materials from cellulose.

## Figures and Tables

**Table 1:** Charge and degree of oxidation (*DO*) or degree of substitution (*DS*) values determined by conductometric titration of TEMPO-oxidized NFC (TOCN5 and TOCN9) and amine-modified NFC (AMCN).

	Charge (mmol g^−1^)	*DO/DS*
TOCN5 (24h)	−0.301 ± 0.113	0.05 ± 0.02
TOCN9 (48h)	−0.531 ± 0.144	0.09 ± 0.02
AMCN	0.227 ± 0.044	0.04 ± 0.01

**Table 2:** Thickness (*d*), charge of the nanopaper, pH stimulus, actuation time (*t_a_*), maximum curvature (*κ_max_*), actuation rate constant (*k*_1_), and actuation rate (*dκ/dt*) for the NFC/TOCN nanopapers.

Film	*d* (µm)	Charge (µmol)	pH	*t_a_* (s)	*κ_max_* (mm^−1^)	*k*_1_ (s^−1^)	*dκ/dt* (mm^−1^ s^−1^)
NFC/TOCN5	52 ± 1	1.6 ± 0.1	high	12	0.071 ± 0.006	0.119 ± 0.020	0.006 ± 0.001
NFC/TOCN9	53 ± 2	2.5 ± 0.0	high	15	0.193 ± 0.024	0.104 ± 0.047	0.013 ± 0.004
NFC/TOCN9	75 ± 1	3.7 ± 0.1	high	23	0.107 ± 0.014	0.038 ± 0.014	0.005 ± 0.001

**Table 3:** Thickness (*d*), pH stimulus, actuation time (*t_a_*), maximum curvature (*κ_max_*), actuation rate constant (*k*_1_), and actuation rate (*dκ/dt*) for the TOCN9/AMCN and the TOCN9/NFC/AMCN nanopapers.

	*d* (µm)	pH	*t_a_* (s)	*κ_max_* (mm^−1^)	*k*_1_ (s^−1^)	*dκ/dt* (mm^−1^ s^−1^)
TOCN9/AMCN	50 ± 0	high	10	0.062 ± 0.006	0.275 ± 0.111	0.009 ± 0.001
TOCN9/AMCN	48 ± 0	low	9	0.056 ± 0.001	0.160 ± 0.017	0.009 ± 0.001
TOCN9/NFC/AMCN	53 ± 0	high	6	0.034 ± 0.001	0.232 ± 0.014	0.006 ± 0.000
TOCN9/NFC/AMCN	45 ± 0	low	10	0.055 ± 0.005	0.210 ± 0.086	0.008 ± 0.000

**Table 4:** pH stimulus, theoretical and effective charges of TOCN9/AMCN and the TOCN9/NFC/AMCN nanopapers.

	pH	Theoretical Charge (µmol)	Effective Charge (µmol)
TOCN9/AMCN	high	2.7 ± 0.0	1.1 ± 0.1
TOCN9/AMCN	low	1.1 ± 0.1	0.7 ± 0.0
TOCN9/NFC/AMCN	high	1.7 ± 0.0	0.4 ± 0.0
TOCN9/NFC/AMCN	low	0.7 ± 0.0	0.7 ± 0.0

## Supplementary Materials

The following are available online at https://www.mdpi.com/2079-4991/10/7/1380/s1. Figure S1: Conductometric titration curve of the TEMPO-oxidized NFC; Figure S2: Characterization of the actuation behavior of NFC films: maximal curvature (*κ_max_*), actuation time (*t_a_*) and actuation rate (*dκ/dt*); Figure S3: Photographs showing the bending of the NFC/TOCN9 films for the thicknesses 55 and 75 µm when they were immersed in high pH (pH 13); and X-Y plots showing the evolution of the bending for the different times from 0 to 60 s; Figure S4: Curvature as a function of time of the TOCN9/NFC/AMCN nanopapers immersed in low and high pH aqueous solutions (pH 1 and 13), respectively; Movie S1: Change in curvature of the NFC/TOCN5 film when it was immersed in high pH; Movie S2: Change in curvature of the NFC/TOCN9 film when it was immersed in high pH; Movie S3: Change in curvature of the TOCN9/AMCN film when it was immersed in high pH; and Movie S4: Change in curvature of the TOCN9/AMCN film when it was immersed in low pH.
